# Effects of biochar and biofertilizer on cadmium-contaminated cotton growth and the antioxidative defense system

**DOI:** 10.1038/s41598-020-77142-7

**Published:** 2020-11-18

**Authors:** Yongqi Zhu, Haijiang Wang, Xin Lv, Yutong Zhang, Weiju Wang

**Affiliations:** College of Agriculture, Shihezi University, Shihezi, 832003 Xinjiang People’s Republic of China

**Keywords:** Physiology, Plant sciences, Environmental sciences

## Abstract

Consistent use of large amounts of fertilizers, pesticides, and mulch can cause the accumulation of harmful substances in cotton plants. Among these harmful substances, cadmium (Cd), an undegradable element, stands out as being particularly highly toxic to plants. The objective of this study was to evaluate the ability of biochar (3%) and biofertilizer (1.5%) to decrease Cd uptake, increase cotton dry weight, and modulate the activities of photosynthetic and peroxidase (POD), superoxide dismutase (SOD), catalase enzyme (CAT) in cotton (*Gossypium hirsutum* L.) grown in Cd-contaminated soil (0, 1, 2, or 4 mg Cd kg^−1^ soil) in pots. These studies showed that, as expected, exogenous Cd adversely affects cotton chlorophyll and photosynthesis. However, biochar and biofertilizer increased cotton dry weight by an average of 16.82% and 32.62%, respectively. Meanwhile, biochar and biofertilizer decreased the accumulation of Cd in cotton organs, and there was a significant reduction in the amount of Cd in bolls (*P* < *0.05*). Biochar and biofertilizer have a positive impact on cotton chlorophyll content, net photosynthesis, stomatal conductance, transpiration rate, and intercellular CO_2_ concentration. Thus, the addition of biochar and biofertilizer promote cotton growth. However, biochar and biofertilizer increased the SOD activity of leaves (47.70% and 77.21%), CAT activity of leaves (35.40% and 72.82%), SOD activity of roots (33.62% and 39.37%), and CAT activity of roots (36.91% and 60.29%), respectively, and the addition of biochar and biofertilizer decreased the content of MDA and electrolyte leakage rate. Redundancy analyses showed that biochar and biofertilizer also improved SOD and POD activities by reducing the heavy metal-induced oxidative stress in cotton and reducing Cd uptake in cotton organs. Therefore, biochar and biofertilizer have a positive effect on the growth of cotton.

## Introduction

Heavy metal pollution is one of the main factors that limit the safety and development of agricultural products grown on soil, and can cause tremendous ecological damage to the environment^1–3^. Heavy metals in soil are derived from two sources: 1) the parent material used to create the soil, and 2) the excessive application of pesticides and fertilizers or irrigation with industrial wastewater and sewage sludge^4,5^. Because its active nature of migration, low critical concentration and easy accumulation of poisoning, Cd is one of the most threatening elements for environment and human health^6,7^, and it has been identified as Class IA carcinogen by International Agency for Research on Cancer. it is also an unnecessary and undegradable element for plants^8,9^. Angelova et al.^10^ showed that the accumulation of Cu, Zn, Pb, and Cd in cotton reached 20.44%, 19.70%, 21.72%, and 10.11%, although the concentration of Cd in cotton is lower than other heavy metals, small dose of Cd is extremely harmful to crops and humans. When it accumulates excessively in the soil, it leads to serious disorders of plant physiology and respiration processes, thus hindering plant growth^11,12^. Therefore, there is a significant need to monitor the effects of cadmium pollution on the physiological characteristics of crops and to identify its mechanisms of toxicity.

A large number of studies have shown that excessive accumulation of Cd in the plant can lead to a series of negative physiological reactions, such as yellowing, wilting, metabolic disorders^13^, and decreasing activity of the photosynthetic system and photosynthetic rate^14–16^. Together, these eventually lead to a reduction of crop yield. For example, when Cd concentration in soil was 0.25 mg·kg^−1^, compared with the control, the chlorophyll content and photosynthetic rate of *C. camphora* decreased by 14.88% and 53.82%, respecitvely^17^. However, the toxic effects of Cd stimulate the production of reactive oxygen species (^1^O_2_, O_2_ and H_2_O_2_), malondialdehyde, and causes electrolyte leakage, leading to significant oxidative stress^18,19^. This reduces cellular metabolism because the production of ROS can induce oxidative stress in the crop, causing the plant to have to produce antioxidant enzymes in order to alleviate the toxicity of ROS^19^.

Recently, there have been reports of the use of metal oxides^20^, rock phosphate^21^, polymeric materials^22^, organic compost^23^, biochar^24^ and microorganisms^25^ to reduce the toxicity of Cd, among which biochar is an environmentally friendly soil amendment with high carbon content, multi-void structure (large surface area), abundant functional groups, strong electronegativity, and other favorable characteristics. Biochar has been shown to increase chlorophyll content, photosynthesis (58.54%), transpiration (59.68%), and stomatal conductance (85%) in plants^26^. It also has antioxidant POD activity (39%)^27^ and CAT activity (36.30%)^28^. Biochar increase resistance against oxidative stress of plants (thus protecting them from heavy metals) and inhibit the absorption of heavy metals by crops. *Bacillus sp.* possess a number of effective metal chelators and functional groups^29^, giving them an impressive ability to absorb heavy metal ions and thus providing notable benefits to the photosynthesis and general quality of crops^30^.

In China, cotton is one of the main cash crops. According to the National Bureau of Statistics, in 2018, the planting area of cotton was 3,352,300 ha, of which most was planted in Xinjiang (2,491,300 ha). The overall average output was 1818.3 kg·ha^−1^. However, with the use of chemical fertilizers, pesticides, and mulch, there has been an enrichment of the heavy metals Cd, Pb, Cu, Zn in the soil^31^ and in all organs of cotton, with the lowest accumulation of Cd in the stems and bolls^32,33^. Therefore, soil supplementation with biochar made from cotton straw could effectively prevent the secondary pollution of soil, and the addition of biofertilizer can be used to increase crop yield, stimulate soil fertility, and prevent pests^34,35^. Biofertilization is less useful for addressing heavy metal pollution in the soil. Therefore, a combination of biochar and biofertilizer was used as amendments to be tested in this study. The main purposes of this work were to: 1) Evaluate the effect of amendments on the accumulation of Cd in different organs of cotton. 2) Explore the effect of amendments and exogenous Cd on oxidative stress reactions, photosynthesis, growth, and development of cotton. 3) Clarify how to improve the negative effects of Cd on cotton by enhancing the antioxidant defense system of cotton in order to produce positive effects on photosynthesis, chlorophyll synthesis, and growth of cotton.

## Results

### Effect of amendments on cotton growth and development

**Figure 1:**
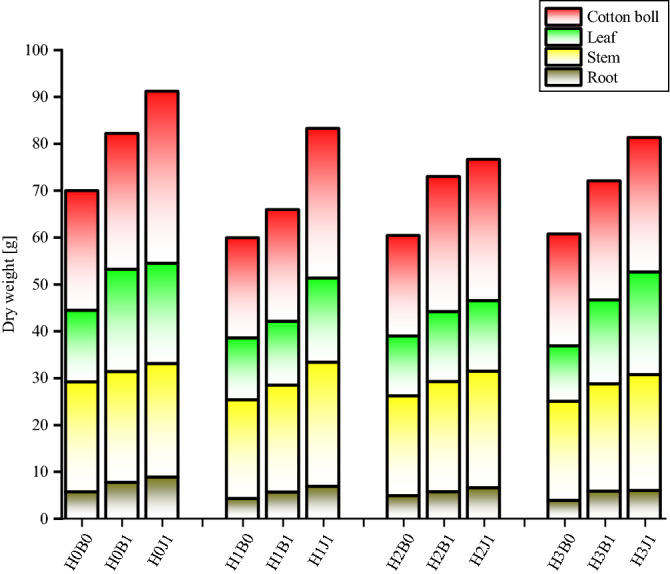
Effect of biochar and biofertilizer on the biomass of different parts of the cotton plant.

Excessive accumulation of the heavy metal Cd can seriously inhibit the growth and development of plants^24,25^. However, these studies suggest that biochar and biofertilizer can significantly rescue this inhibition (Fig. 1). Compared with control H0B0 treatment, H1B0, H2B0, and H3B0 treatments reduced the total dry weight of cotton of 16.86%, 15.89% and 15.33%, respectively (See Table 2 for treatment conditions). The dry weight of cotton decreased gradually with increasing Cd concentration. After the application of biochar and biofertilizer, however, the dry weight of cotton organs increased. After treatment with H0B1, the dry weights of roots and stems were increased by 37.96% and 0.58%, respectively, and the dry weights of leaves and bolls increased by 43.27% and 13.24%, respectively, compared with H0B0 (negative control). After treatment with H0J1, there was an increase in the dry weight of roots (58.01%), stems (3.22%), bolls (43.64%), and leaves (40.11%) of cotton. The maximum dry weight of cotton was 91.17 g, obtained after treatment with H0J1 treatment. When the content of heavy metal added was 1, 2, and 4 mg·kg^−1^, the effect of the amendments on the dry weight of cotton had a similar trend.

### Effect of biochar and biofertilizer on Cd absorption and transportation in cotton

The cotton root is the main enrichment site of Cd (Table 1), with a maximum accumulation of 0.291 mg·kg^−1^, followed by leaves and stems, and last by the bolls. With an increase of exogenous Cd addition, the accumulation of Cd in all organs of cotton increased, as expected. Compared with the control H0B0, the accumulation of Cd in the roots, leaves, stems, and bolls of cotton treated with H2B0 and H3B0 increased by 17.39% and 40.58%, 11.32% and 35.85%, 28.86% and 32.21%, 15.53% and 31.07% *(P* < *0.05),* respectively. Compared with no amendments, biochar and biofertilizer supplementation significantly reduced the accumulation of Cd in cotton and significantly affected the accumulation of Cd in bolls *(P* < *0.05)*, and there was a significant difference between the amendments *(P* < *0.05)*. For example, in the biochar (H3B1) and biofertilizer (H3J1) treatments, the Cd content in the roots, leaves, stems, and bolls of cotton decreased by 20.27% and 17.87%, 4.63% and 11.11%, 15.23% and 15.74, 26.67% and 8.89%, respectively, relative to plants treated with H3B0. In line with our hypothesis, the regression tests results showed that exogenous Cd, amendments, and the interaction between amendments and Cd had a significant or extremely significant effect on the accumulation of Cd in cotton organs *(P* < *0.05)*.

**Table 1:** Effect of biochar and biofertilizer on contents of Cd in roots, stems, leaves, and bolls of cotton.

Cd (mg kg^−1^)	Amendments (%)	Cd content			
		Root	Leaf	Stem	Cotton boll
H0	B0	0.0207 ± 0.001 d	0.0159 ± 0.016 f.	0.0149 ± 0.007 cd	0.0103 ± 0.005 c
	B1	0.0178 ± 0.017 e	0.0109 ± 0.011 g	0.0089 ± 0.004 h	0.0075 ± 0.003 e
	J1	0.0175 ± 0.017 e	0.0109 ± 0.011 g	0.0109 ± 0.005 g	0.0087 ± 0.004 d
H1	B0	0.0214 ± 0.021 cd	0.0170 ± 0.017 def	0.0159 ± 0.007 bc	0.0106 ± 0.005 c
	B1	0.0203 ± 0.020 d	0.0159 ± 0.016 f.	0.0122 ± 0.006 f.	0.0078 ± 0.004 e
	J1	0.0203 ± 0.020 d	0.0150 ± 0.018 f.	0.0123 ± 0.006 f.	0.0050 ± 0.003 f.
H2	B0	0.0243 ± 0.024 b	0.0177 ± 0.018 de	0.0192 ± 0.009 a	0.0119 ± 0.005 b
	B1	0.0227 ± 0.023 bc	0.0172 ± 0.017 def	0.0132 ± 0.006 ef	0.009 ± 0.004 d
	J1	0.0228 ± 0.023 bc	0.0162 ± 0.016 ef	0.0138 ± 0.006 de	0.0099 ± 0.004 c
H3	B0	0.0291 ± 0.029 a	0.0216 ± ± 0.22 a	0.0197 ± 0.009 a	0.0135 ± 0.006 a
	B1	0.0232 ± 0.023 bc	0.0206 ± 0.21 ab	0.0167 ± 0.008 b	0.0099 ± 0.004 c
	J1	0.0239 ± 0.021 b	0.0192 ± 0.19 bc	0.0166 ± 0.008 b	0.0123 ± 0.006 b
Regression tests (significance)					
H		**	**	**	**
BJ		**	**	**	**
BJ*H		*	**	**	**

Values show the means of five replicates ± SE. Different lowercase letters in the same column indicate significant differences (*P* < *0.05*) in Cd content among individual treatments. **, *P* < *0.01;* ns, *P ≥ 0.05.*

The effects of amendments on the Cd transfer coefficient to different parts of cotton plant are shown in Table 2. These studies found that, under the same treatment, F2 (Cd transportation from stem to leaf) > F1 (root to stem) and F3 (stem to boll), but that the application of biochar and biofertilizer significantly reduced the migration of Cd from root to stem *(P* < *0.05)*, For example, in the H2B1 and H2J1 treatments, the value of F1 (originally 0.079 with H2B0 treatment) decreased to 0.058 and 0.061, respectively. In contrast, the two amendments increased the migration of Cd from stems to leaves.

**Table 2:** Effect of biochar and biofertilizer on transportation coefficients of Cd in cotton.

Treatments	F1 (root-stem)	F2 (stem-leaf)	F3 (stem-cotton boll)
H0B0	0.72 ± 0.03 bc	1..07 ± 0.04 de	0.69 ± 0.03 bcd
H0B1	0.50 ± 0.02 f.	1.22 ± 0.05 bc	0.84 ± 0.03 a
H0J1	0.62 ± 0.03 de	1.00 ± 0.04 ef	0.80 ± 0.03 a
H1B0	0.74 ± 0.03 ab	1.07 ± 0.04 de	0.67 ± 0.03 cde
H1B1	0.60 ± 0.02 e	1.30 ± 0.05 b	0.64 ± 0.03 edf
H1J1	0.61 ± 0.02 e	1.46 ± 0.06 a	0.08 ± 0.02 g
H2B0	0.79 ± 0.03 a	0.92 ± 0.04 f.	0.62 ± 0.03 ef
H2B1	0.58 ± 0.02 e	1.30 ± 0.05 b	0.68 ± 0.03 bcd
H2J1	0.61 ± 0.02 e	1.17 ± 0.05 cd	0.72 ± 0.03 bc
H3B0	0.72 ± 0.03 bc	1.10 ± 0.04 de	0.69 ± 0.03 bcd
H3B1	0.68 ± 0.03 cd	1.23 ± 0.05 bc	0.59 ± 0.02 f.
H3J1	0.69 ± 0.03 bc	1.16 ± 0.05 cd	0.74 ± 0.03 b

Values show the means of five replicates ± SE. Means followed by same small letters are not significant different at *P* < *0.05* by using the Duncan test.

### Effect of amendments on the chlorophyll content of cotton

Chlorophyll is a pigment crucial for light capture and photosynthesis^36^. The changes in chlorophyll content are shown in Table 3. These studies found that the content of chlorophyll was reduced by adding exogenous Cd without biochar or biofertilizer. Indeed, compared with H0B0 treatment, the content of chlorophyll a, chlorophyll b, and carotenoid reduced by 1.55%, 2.13%, and 1.37% after H1B0 treatment, respectively, and after treatment with H2B0, decreased by 6.51%, 4.35%, and 1.37%, respectively. After treatment of H3B0, these values decreased by 22.43%, 29.73%, and 7.25%, respectively. However, the application of biochar and biofertilizer significantly increased the content of chlorophyll a and b *(P* < *0.05)*, and there was a significant difference between the amendments on the content of chlorophyll a *(P* < *0.05)*. In the H3B1 and H3J1treatments, the content of chlorophyll a (42.98% and 69.84%) and chlorophyll b (31.92% and 37.55%) experienced the greatest increase, and chlorophyll a/b followed a similar trend. The amendments obviously increased the content of carotenoid, and biofertilizer had a significant effect on the content of carotenoid relative to control plants *(P* < *0.05)*. After treatments with H0B1 and H0J1, compared with H0B0, the content of carotenoids increased most dramatically, by 18.44% and 31.56%, respectively. The results of regression tests showed that exogenous Cd had a significant or extremely significant effect on chlorophyll b and carotenoids *(P* < *0.05)*, however the interaction of amendments or amendments and exogenous Cd had no significant effect on chlorophyll content *(P* < *0.05).*

**Table 3:** Effect of biochar and biofertilizer on photosynthetic pigment contents in cotton leaves.

Cd (mg kg^−1^)	Amendments (%)	Photosynthetic pigment contents (mg kg^−1^ FW)			
		Chl a	Chl b	Chl a/b	Car
H0	B0	1.31 ± 0.05 f.	0.48 ± 0.02 d	2.71 ± 0.74 e	0.74 ± 0.03 de
	B1	1.84 ± 0.08 bc	0.57 ± 0.02 cd	3.22 ± 0.88 b	0.88 ± 0.04 b
	J1	2.02 ± 0.08 a	0.63 ± 0.03 a	3.21 ± 0.97 bc	0.97 ± 0.04 a
H1	B0	1.29 ± 0.05 f.	0.47 ± 0.02 d	2.74 ± 0.73 e	0.73 ± 0.03 de
	B1	1.70 ± 0.07 cd	0.55 ± 0.02 c	3.08 ± 0.86 bcd	0.86 ± 0.04 bc
	J1	1.89 ± 0.08 ab	0.61 ± 0.03 ab	3.09 ± 0.87 bcd	0.87 ± 0.04 b
H2	B0	1.23 ± 0.05 f.	0.46 ± 0.02 d	2.66 ± 0.73 e	0.73 ± 0.03 de
	B1	1.58 ± 0.06 de	0.55 ± 0.02 c	2.87 ± 0.79 de	0.79 ± 0.03 cd
	J1	1.85 ± 0.08 b	0.57 ± 0.02 c	3.27 ± 0.80 b	0.80 ± 0.03 cd
H3	B0	1.07 ± 0.04 g	0.37 ± 0.01 e	2.93 ± 0.69 cde	0.69 ± 0.03 e
	B1	1.54 ± 0.06 e	0.48 ± 0.02 d	3.18 ± 0.72 bc	0.72 ± 0.03 de
	J1	1.82 ± 0.07 bc	0.50 ± 0.02 d	3.62 ± 0.77 a	0.77 ± 0.03 d
Regression tests (significance)					
H		ns	*	ns	**
BJ		ns	ns	ns	ns
BJ*H		ns	ns	ns	ns

Values show the means of five replicates ± SE. Different lowercase letters in the same column indicate significant differences (*P* < *0.05*) in photosynthetic pigment contents among individual treatments. **, *P* < *0.01*; ns, *P ≥ 0.05.*

### Effect of amendments on photosynthesis of cotton

Understanding the photosynthetic response of plants is central to understanding the physiological response^37^. Table 4 shows the effects of amendments and Cd on photosynthetic parameters of cotton. Compared with the H0B0 treatment, H1B0, H2B0, and H3B0 significantly reduced the net photosynthetic rate, stomatal conductance, intercellular CO_2_ concentration, and transpiration rate of cotton *(P* < *0.05)*. Further, the net photosynthetic rate, stomatal conductance, and transpiration rate were significantly different across groups treated with H1B0, H2B0, and H3B0 *(P* < *0.05)*, but there was no significant difference in the intercellular CO_2_ concentration. The application of biochar and biofertilizer increased the net photosynthetic rate, stomatal conductance, intercellular CO_2_ concentration, and transpiration rate, and there were significant differences in the H0B1, H0J1and H1B1, H1J1 treatments compared with treatments with H0B0 or H1B0 *(P* < *0.05)*. Compared with no amendments, biochar led to a maximum increase in the net photosynthetic rate (26.11%), stomatal conductance (268.7%), intercellular CO_2_ concentration (92.65%), and transpiration (203.6%), Compared with no amendments, biofertilizer led to an maximum increase in the net photosynthetic rate (112.60%), stomatal conductance (32.92%), intercellular CO_2_ concentration (92.65%), and transpiration (128.20%).

**Table 4:** Effect of biochar and biofertilizer on photosynthetic pigment contents in cotton.

Cd (mg kg^−1^)	Amendments (%)	Net photosynthetic rate (µmol m^−2^ s^−1^)	Stomatal conductance (mmol m^−2^ s^−1^)	Intercellular CO_2_ concentration (µmol mol^−1^)	Transpiration rate (mmol m^−2^ s^−1^)
H0	B0	17.62 ± 0.72 d	0.18 ± 0.007 e	430.85 ± 17.59 cd	6.53 ± 0.27 d
	B1	20.03 ± 0.82 bc	0.33 ± 0.014 a	605.40 ± 24.72 a	12.61 ± 0.51 a
	J1	23.24 ± 0.95 a	0.24 ± 0.009 c	513.31 ± 20.96 b	9.37 ± 0.37 c
H1	B0	15.42 ± 0.63 e	0.09 ± 0.004 f	387.49 ± 15.82 e	3.31 ± 0.14 f
	B1	19.45 ± 0.79 c	0.30 ± 0.012 b	595.10 ± 24.29 a	10.06 ± 0.41 b
	J1	21.16 ± 0.86 b	0.09 ± 0.004 f	447.93 ± 18.29 c	5.11 ± 0.21 e
H2	B0	9.17 ± 0.37 g	0.06 ± 0.003 g	223.47 ± 9.12 h	3.03 ± 0.12 e
	B1	10.24 ± 0.42 g	0.23 ± 0.009 d	430.52 ± 17.57 cd	6.95 ± 0.28 d
	J1	14.69 ± 0.60 e	0.06 ± 0.003 g	349.77 ± 14.28 f	4.89 ± 0.20 f
H3	B0	6.21 ± 0.25 h	0.02 ± 0.009 h	241.99 ± 9.88 h	1.39 ± 0.06 g
	B1	7.53 ± 0.31 h	0.07 ± 0.003 g	396.05 ± 16.17 de	3.61 ± 0.15 f
	J1	13.20 ± 0.54 f	0.06 ± 0.003 g	302.53 ± 12.35 g	3.16 ± 0.13 f
Regression Tests (significance)					
H		**	**	**	**
BJ		ns	ns	ns	ns
BJ*H		ns	ns	ns	ns

Values show the means of five replicates ± SE. Different lowercase letters in the same column indicate significant differences (*P* < *0.05*) in photosynthetic pigment contents among individual treatments. **, *P* < *0.01*; ns, *P ≥ 0.05*.

### Effects of amendments and exogenous Cd on the activity of antioxidant enzymes and the electrolyte leakage rate of cotton

**Figure 2:**
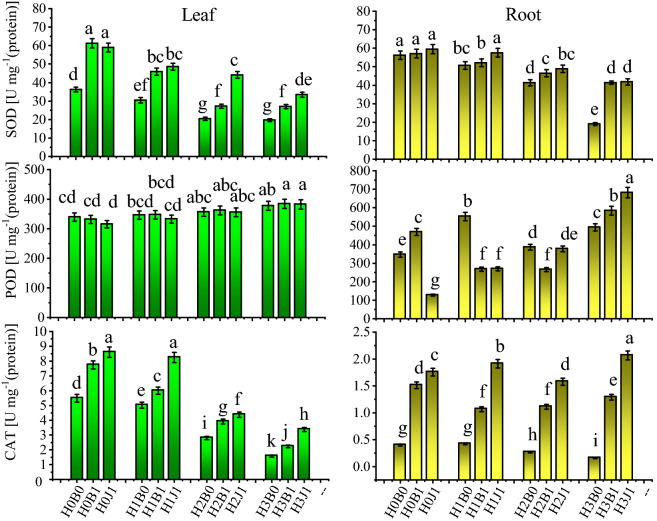
Effect of biochar and biofertilizer on activity of antioxidant enzymes in leaf and root of cotton plants. Values show the mean of five replicates ± SE. Means followed by same small letters are not significant different at *P* < 0*.*05 by using the Duncan test.

The cotton plant has an antioxidant system that can remove harmful substances in the plant to protect the cells from oxidative damage^38^. As shown in Fig. 2, compared with H0B0, there were significant differences in SOD and CAT activities of leaves, SOD and POD activities of roots in the H1B0, H2B0, and H3B0 treatments *(P* < *0.05)*. Compared with no amendments, the application of biochar and biofertilizer significantly increased the SOD and CAT activities of leaves, and CAT activity of roots *(P* < *0.05)*. Compared with no amendments, the maximum increase in the activity of SOD and CAT in leaves with biochar alone followed treatment with H0B1 (68.97% and 40.80%, respectively), and the maximum increase in activity of SOD and CAT in leaves treated with biofertilizer was with H3J1 (113.9% and 70.29%, respectively). The maximum increases in SOD were observed after treatments with H3B1 and H3J1, which increased SOD activity by 117.6% and 119.8%, respectively. CAT activity in the roots also showed similar trends. However, there was no obvious effect on POD after treatment with amendments.

**Figure 3:**
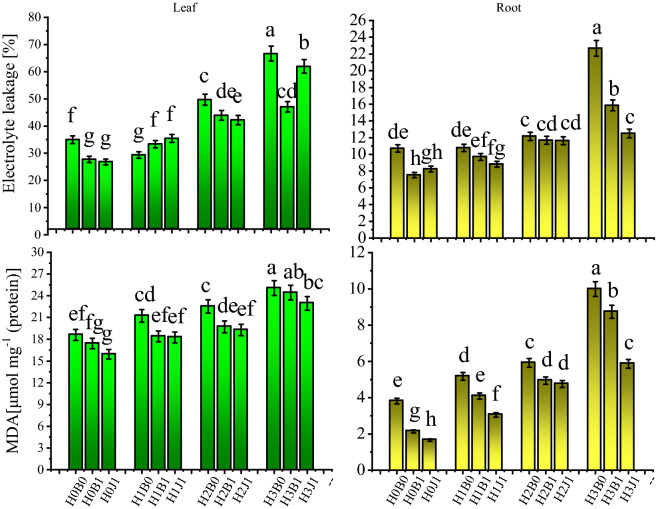
Effect of biochar and biofertilizer on malondialdehyde (MDA) and electrolyte leakage (EL) in the leaf and root of cotton plants. Values show the means of five replicate ± SE. Means followed by the same small letters are not significantly different at *P* < 0*.*05 by using the Duncan test.

The effects of amendments and heavy metals on MDA and the electrolyte leakage rate in the leaves and roots are shown in Fig. 3. Exogenous Cd significantly increases MDA content and the electrolyte leakage rate. After exogenous Cd addition, the MDA content and electrolyte leakage rate were increased. The maximum MDA content of leaves and roots was 25.045 and 9.994 μ mol·mg^−1^ after H3B0 treatment, and the maximum electrolyte leakage rate of leaves and roots was also observed after H3B0 treatment. Compared with no amendments, biochar and biofertilizer significantly decreased MDA content of roots and the electrolyte leakage rate of leaves *(P* < *0.05)*. The maximum decrease in MDA content and electrolyte leakage in the leaves was observed for biochar after treatments with H1B1 and H3B1. Compared with H1B0 and H3B0, the application of biochar led to decrease in MDA and the electrolyte leakage rate in the leaves of 13.39% and 29.34%, respectively. MDA content and the electrolyte leakage rate in the cotton leaves were most dramatically decreased after treatments with H2J1 and H1J1, by 14.42% and 13.98%, respectively.

### Redundancy analysis between cotton growth index and environmental factors

**Figure 4:**
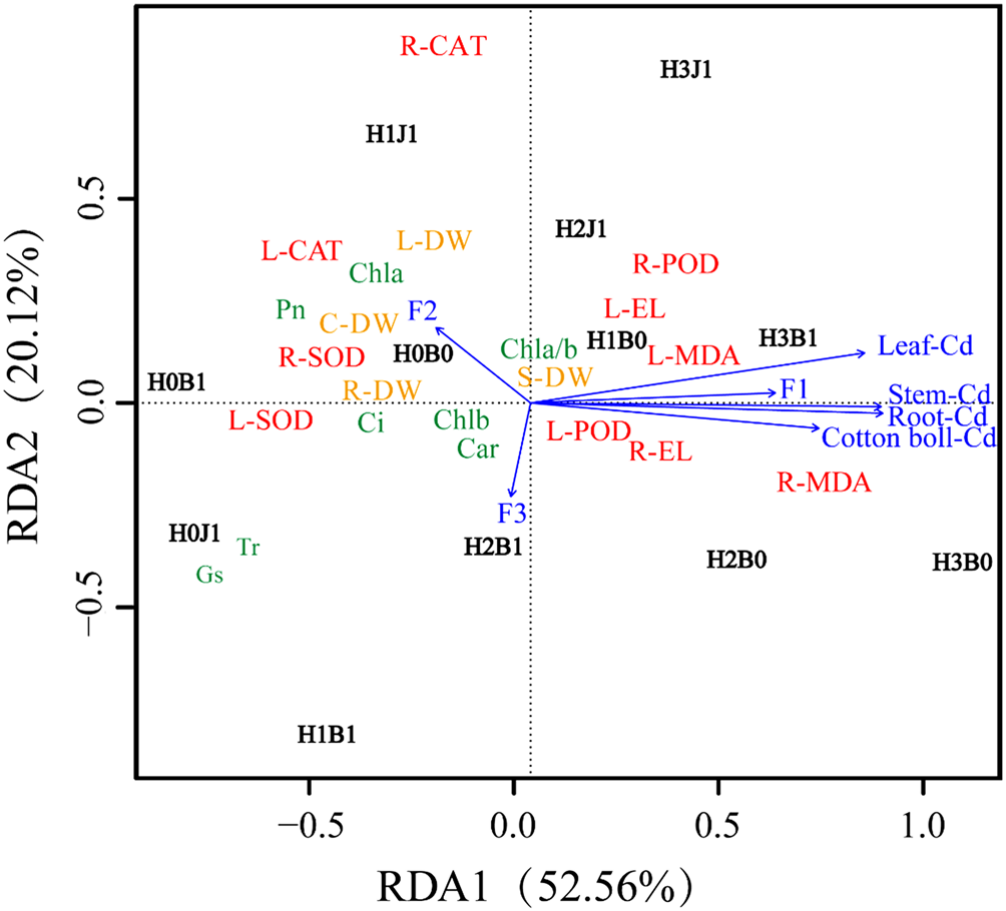
Redundancy analysis of cotton growth index and physiological characteristics. L,R-SOD, L,R-POD, L,R -CAT, L,R -MDA, L,R -EL represents leaves and roots SOD, POD, CAT, MDA, electrolyte leakage rate, DW represents dry weight, Tr representative transpiration ratio, Gs represents stomatal conductance, Pn represents net photosynthesis rate, Ci represents intercellular CO_2_ concentration.

Through redundancy analysis (Fig. 4), the relationship between Cd absorption, transport in cotton organs, cotton growth, and physiological indicators were analyzed. The first principal component accounted for 52.56%, the second principal component accounted for 20.12%, and the cumulative rate was 72.68%, which could explain all variables. The results showed that the treatments were rather variable, suggesting that the differences of outcomes under each treatment were larger. The Cd arrows in cotton leaves, stems, and roots were longer, indicating that Cd content in cotton leaves, stems, and roots had a greater impact on the physiological indicators. The Cd content of cotton was closely related to POD, electrolyte leakage rate, and MDA of roots and leaves, which were distributed in the first and fourth quadrants, and H1B0, H2B0, and H3B0 were also distributed in the first and fourth quadrants, indicating that Cd treatment has a strong influence on Cd content in cotton, as would be expected. In the second and third quadrants, the growth indexes (dry weight, photosynthesis, and chlorophyll content) of cotton were closely related to POD and SOD of leaves and roots and negatively related to Cd of cotton.

## Discussion

Cadmium ranks as the fourth most harmful element for plants, and extremely inhibits the growth and development of plants^39^. Biochar, however, can improve the stability of Cd, alleviate Cd-induced stress for plants, and enhance fertility to promote the growth and development of plants^40^. Biofertilizer is comprised of a large number of living bacteria and nutrient elements, and has a significant positive effect on the ecological environment of soil and the growth and development of crops^41,42^. Recently, biofertilizer has been widely reported as a biosorption agent for heavy metals in soil^42,43^. In this study, the addition of exogenous Cd decreased the dry weight of cotton, at the highest dose of exogenous Cd (4 m·kg^−1^), the total dry weight of cotton was the lowest (60.63 g). However, treatment with biochar and biofertilizer increased the dry weight of each organ of cotton and promoted growth and development (Fig. 1), in good agreement with the findings of Grzesik et al.^44^. The increase in cotton biomass is because biochar and biofertilizer contain a large number of nutritive elements which can provide a favorable growth environment for plants^45,46^.

In this study, the cotton root was the main site of enrichment site for Cd uptake and the cotton stem and boll had the lowest accumulation of Cd, in line with the results of Chen et al.^47^. Biochar and biofertilizer can effectively reduce the concentration of Cd in cotton organs and significantly reduce the content of Cd in the cotton stem and boll *(P* < *0.05)*. Biochar and biofertilizer can also reduce the transportation of Cd from root to stem (Tables 1, 2), thus reducing the accumulation of Cd in different organs of cotton, which is consistent with the results of Yang et al.^32^ and Bharwana et al.^33^. Biochar can reduce the transportation of Cd by surface complexation, precipitation, and ion exchange^48^. *Bacillus* strains isolated in the study of Ka-Ot et al.^49^. have strong tolerance to 800 mg·L^−1^ Cd, probably because they can accumulate Cd at the glutamic acid carboxyl of peptidoglycan, which is associated with a large number of granular Cd deposits in the cell wall^50^. The use of intracellular and extracellular sequestration may be an important way to promote cadmium resistance^29^, and reduce the accumulation of Cd in plants.

Photosynthesis is an extremely important physiological process in plants that plays a decisive role in growth and development^48^. However, under Cd stress, photosynthetic pigments in plants are decreased^51,52^, the number of stomata in the leaves are decreased^53^, and there are thus negative effects on the net photosynthetic rate, stomatal conductance, intercellular CO_2_ concentration, and transpiration rate^54^. In this study, the addition of exogenous Cd obviously reduced the contents of chlorophyll a (22.43%), chlorophyll b (29.73%) and carotenoids (7.25%) (Table 3). The decrease of photosynthetic pigments may be due to the production of a large amount of hydrogen peroxide in the plant or the enzymatic degradation of chlorophyll by an enzyme^55^. Treatment with exogenous Cd significantly decreased net photosynthesis, stomatal conductance, intercellular CO_2_ concentration, and transpiration rate *(P* < *0.05)*, with maximum reductions of 67.76%, 88.89%, 43.83%, and 78.71% compared with the untreated control, respectively (Table 4), which was consistent with the results of Ci et al.^56^. Krantev et al.^57^ showed that the decrease of photosynthesis was related to the decrease of carboxylase, phosphoenolpyruvate carboxylase, and ribose 1, 5-diphosphate carboxylase activities under Cd stress. The application of biochar and biofertilizer, however, significantly increased the content of chlorophyll a and chlorophyll b *(P* < *0.05)* (Table 3), and also enhanced the net photosynthetic rate, stomatal conductance, intercellular CO_2_ concentration, and transpiration rate in cotton (Table 4). Biochar and biofertilizer can effectively enhance the stability of heavy metals in soil, alleviating the inhibitory effects of Cd on photosynthesis and chlorophyll production^27,50^.

Under Cd stress, a large amount of ROS are produced, and those ROS can greatly damage the physiology and metabolism of plants. The mechanism that eliminates ROS is based on the combined action of superoxide dismutase and catalase^27^, thus protecting the cell structures from damage^58^. In addition, the toxicity of Cd is related to the free radicals in membrane components, which decrease the movement and permeability of free ions in plant tissues^59^, generate a large amount of MDA, and then induce oxidative stress in plants^60^. In this study, the addition of exogenous Cd decreased the activity of SOD and CAT in leaves and roots and increased the electrolyte leakage rate and MDA content (Figs. 2, 3), which was consistent with the results of Muszyńska et al.^61^ and Roychoudhury et al.^62^. However, the application of biochar and biofertilizer increased the activities of antioxidant enzymes and increased the oxidative stress response of plants to Cd^24,25,43,48^. In this study, biochar and biofertilizer increased the activities of SOD and CAT and reduced the content of MDA and electrolyte leakage rate, effectively alleviating the negative effects of Cd in cotton (Figs. 2, 3). This is mainly because CAT activity is responsible for scavenging the effects of toxic peroxides in plant cells, and superoxide dismutase (SOD) is the key enzyme for the decomposition of superoxide radicals into H_2_O_2_. There are three different subtypes: CuZn-SOD, Mn-SOD, and Fe-SOD^63^. POD activity in plants is responsible for respiratory metabolisms and the transformation of phenols into quinones to reduce the toxicities of heavy metal-induced oxidative stress^64^. The improvement of antioxidant defense system was mainly due to amendments that can improve plant health and eliminate active oxygen so that the plant can resist metal stress. In addition, the reduction of Cd in the organs of plants likely enhances anti-ROS protection in plants.

These results were confirmed by RDA analysis. The content of Cd in cotton organs was negatively correlated with the content of SOD, CAT, chlorophyll, and photosynthetic parameters of leaves and roots, indicating that the accumulation of Cd in cotton organs greatly inhibited the physiological indexes of cotton. Indeed, the content of Cd in cotton organs was positively correlated with MDA and electrolyte leakage of leaves and roots. In this study, biochar and biofertilizer enhanced the antioxidant mechanisms of cotton to reduce the accumulation of Cd, attenuate the toxic effects of Cd on cotton, and ultimately cause a positive effect on the growth and development of cotton flowers.

## Conclusion

Biochar and biofertilizer can effectively reduce the accumulation of Cd in cotton organs and significantly reduce the migration of Cd from roots to stems *(P* < *0.05)*. Biochar and biofertilizer demonstrated an impressive ability to inhibit the absorption of Cd by cotton stems. Under different levels of exogenous Cd, the accumulation of Cd in stems reduced by 27.50% and 25.14% on average after applying biochar and biofertilizer, respectively. The accumulation of exogenous Cd inhibited the synthesis of chlorophyll, photosynthesis, and antioxidant enzyme activity in cotton, ultimately inhibiting growth and development. However, the application of biochar and biofertilizer can help defend against the toxic mechanisms of Cd to restore growth and development of cotton. Therefore, these findings suggest a role for biochar and biofertilizer as a method to address repair heavy metal pollution.

## Materials and methods

### Collection and physicochemical properties of soil

The soil samples were collected from soil from a local cotton field. Before collecting the samples, residue was removed, it was air dried, and then passed through a 5-mm sieve. The basic physical and chemical properties of soil (Table 5), such as soil pH (soil water content [1:2.5 of W / V]), soil conductivity (calculated by a calibrated conductivity meter [BANTE, DDS-12DW, China] at 25 °C, soil organic matter (via oil bath method), soil total nitrogen (Kjeldahl method), soil total phosphorus (Cary 60 ultraviolet spectrophotometer), total potassium^65^, and total Cd measurement (hydrochloric acid : nitric acid (ratio 3:1) using an atomic absorption spectrophotometer^24^.

**Table 5:** Basic physical–chemical properties of biochar and soil used in the experiments.

Property	Biochar	Soil
pH	9.50	7.76
Total nitrogen (g kg^−1^)	0.89	0.46
Total P (g kg^−1^)	2.54	28.42
Organic matter (g kg^−1^)	625	14.73
Total K (g kg^−1^)	8.62	246.83
Total Cd (mg kg^−1^)	0.002	0.25
Total salinity (g kg^−1^)	−	3.36
Carboxyl (mmol g ^−1^)	0.20	−
Lactone (mmol g ^−1^)	0.25	−
Phenolic hydroxyl (mmol g^−1^)	0.21	−

### Preparation and analysis of amendments

The biochar (B) in this experiment was cotton straw biochar, and it was produced as previously described^28^. The basic physical and chemical properties of biochar are shown in Table 5. Biochar was air dried, passed through a 5-mm sieve, and then basic physical and chemical properties were measured, including pH, organic, total nitrogen, total phosphorus, total potassium, and total Cd^66^. Biofertilizer (J) was purchased from a company in Shandong Province, China. The functional bacteria were *Bacillus* composite. The basic physical and chemical properties of J were measured according to the standards of microbial pathogens in agriculture (SMIA, national standard of China, GB 20287-2006). The number of total living bacteria was ≥ 20 billion·g^−1^, the bacterial mixture was > 99.6% Bacillus was < 0.4%, the water content was < 10%, the pH was 7.8, and the total Cd content was 0.0001 mg·kg^−1^.

### Preparation of contaminated soil

In this study, exogenous CdCl_2_·2.5 H_2_O (2.44 g, analytical purity) was dissolved in distilled water, shaken well, and diluted into 1000 ml to obtain 1.2 g·L^−1^ of Cd^2+^ solution. Then, 10 ml, 20 ml, or 40 ml of this solution were mixed with 12 kg soil samples to produce 1, 2, 4 mg·kg^−1^ of exogenous Cd^2+^ test samples. These levels are equivalent to 3, 6, and 11 times of the global average content of Cd in soil^67,68^. The contaminated soil as preserved for 60 days for subsequent tests^69^.

### Experimental design

In this study, the polluted soil was mixed with 3% (w / w, 46.8 t·ha^−1^) biochar^8^ and 1.5% (w / w, 4.5 kg·ha^−1^) biofertilizer^41^ and then stored in a plastic flowerpot (25 cm × 40 cm) for one week at room temperature (25 °C). In this experiment, a complete randomized design was used. Twelve treatments (five replicates for each treatment) were setup (Table 6). The soil was irrigated with deionized water and maintained at 60% of the field water content. The type and amount of fertilizer applied to each pot was N-P_2_O_2_-K_2_O (180–150–210 kg·hm^2^ urea, diammonium phosphate and potassium sulfate). All phosphorus and potassium, and half of the nitrogen were applied before sowing, and the rest was applied after the crop was planted.

**Table 6:** Addition amount of Cd, biochar and biofertilizer in each treated soil.

Treatments	Cd (mg kg^−1^)	Biochar (%)	Biofertilizer (%)
H0B0	0	0	0
H0B1	0	3%	0
H0J1	0	0	1.5%
H1B0	1	0	0
H1B1	1	3%	0
H1J1	1	0	1.5%
H2B0	2	0	0
H2B1	2	3%	0
H2J1	2	0	1.5%
H3B0	4	0	0
H3B1	4	3%	0
H3J1	4	0	1.5%

### Cotton planting and management

The cotton seeds (*Gossypium hirsutum* L.) used in this experiment were purchased from the local market. The variety was "Xinluzao 53." Seeds of the same size were selected. The seeds were sterilized with 2.5% sodium hypochlorite solution, and 20 seeds were planted in each pot. After 3 true leaves of cotton were grown, 5 seedlings with the same apparent growth were selected for cultivation. In order to prevent secondary pollution, deionized water was used for irrigation. Plant samples were collected at the stage of cotton bolls. We collected cotton roots, stems, leaves, and bolls, washed them with deionized water, weighed them on a digital scale, determined the fresh weight of each sample, and first used the oven to kill the plants (105 °C, 2 h) and then dry (85 °C) to a constant dry weight.

### Test indicators and methods

Next, we accurately weighed 0.5 g of dried plants (roots, stems, leaves, and bolls), sealed the samples under high pressure and temperature, and digested them with a mixture of nitric acid: perchloric acid (2:1), according to a previously described method^66^. Then, we determined the total content of Cd in the roots, stems, leaves, and bolls using an atomic absorption spectrophotometer. The formula for the transfer coefficient is as follows:

1 $$ {\text{Transfer}}\,{\text{coefficient}}\,\left( {{\text{F}}1} \right) = \frac{{{\text{Stems}}\,{\text{Cd}}}}{{{\text{Roots}}\,{\text{Cd}}}} $$

2 $$ {\text{Transfer}}\,{\text{coefficient}}\,\left( {{\text{F}}2} \right) = \frac{{{\text{Leaves}}\,{\text{Cd}}}}{{{\text{Stems}}\,{\text{Cd}}}} $$

3 $$ {\text{Transfer}}\,{\text{coefficient}}\,\left( {{\text{F}}3} \right) = \frac{{{\text{Bools}}\,{\text{Cd}}}}{{{\text{Stems}}\,{\text{Cd}}}} $$

Determination of antioxidant enzymes in leaves and roots.

The activity of antioxidant enzymes in leaves was determined by spectrophotometry. The activity of superoxide dismutase (SOD) was determined according to the method of Paoletti et al.^70^ based on photochemical reduction of NBT. The activities of catalase (CAT) and peroxidase (POD) were determined as described by Cakmak and Marschner^71^. The content of malondialdehyde (MDA) in leaves was determined by TBARS^33^. Electrolyte leakage was determined as previously described^33^.

Determination of chlorophyll and photosynthetic characteristics.

Chlorophyll was extracted with 80% acetone and compared at 663.2, 646.5, and 470 nm. The content of chlorophyll (Chla and Chlb) and carotenoid (Car) were calculated using the Lichtenthaler^72^ equation. Net photosynthetic rate (PN), stomatal conductance (GS), transpiration rate (TR), and intracellular CO_2_ concentration (CI) were measured using a Li-6400 portable photosynthetic system.

The data were compiled in Excel 2016 and regression tests was performed using SPSS 23.0. Multiple comparisons between different treatments were conducted using Duncan's new multiple range test (α = 0.05). Charts were drawn using Origin 8.0 (OriginLab, MA, USA).
